# Pingchuan formula improves asthma via restoration of the Th17/Treg balance in a mouse model

**DOI:** 10.1186/s12906-015-0755-8

**Published:** 2015-07-16

**Authors:** Fei Liu, Jianer Yu, Li Bai, Zheng Xue, Xinguang Zhang

**Affiliations:** Pediatrics Shanghai Academy, Shanghai Municipal Hospital of Traditional Chinese Medicine affiliated to Shanghai TCM University, Shanghai, 200071 China; Wuxi Municipal Hospital of Traditional Chinese Medicine, Wuxi, 214001 China

**Keywords:** Th17/Treg, Asthma, Pingchuan formula (PCF), Asthma mouse model

## Abstract

**Background:**

Pingchuan Formula (PCF) is a traditional Chinese recipe. PCF improves chronic airway inflammation by correcting the imbalance of T-helper cell ratio. The purpose of this study was to investigate the effect of PCF on pathological changes in the lungs of asthmatic mice in terms of Treg/Th17 balance.

**Methods:**

A bronchial asthma BALB/c mouse model was established using the ovalbumin excitation method. Distilled water (for MDL group) and drugs (for DEX or PCF group) were administered by gavage immediately after the first excitation. Mice were sacrificed after 7 and 28 d treatment. Lung tissues and bronchoalveolar lavage fluid were collected and lung pathological changes were observed after hematoxylin and eosin staining. Differential cell counts, concentrations of interleukins-6, -17, -23 and TGF-β in bronchoalveolar lavage fluid were determined by enzyme-linked immunosorbent assay. Expression of transcriptional factors Foxp3 and RORγt was determined by immunohistochemistry and immunoblot.

**Results:**

An asthma model was successfully established. After 7 or 28 d treatment, lung pathological changes were improved and concentration of interleukins-6, -17, -23 and TGF-β in bronchoalveolar lavage fluid significantly decreased in the PCF group. RORγt expression in lung tissue was decreased in the PCF group, while Foxp3 expression increased (all P values < 0.05 compared with the MDL group). There was no significant difference between the PCF and DEX group except that mice in the PCF group lost less bodyweight.

**Conclusions:**

Treatment with PCF downregulates RORγt, elevates Foxp3 expression, reduces interleukins-6, -17, -23 and TGF-β in bronchoalveolar lavage fluid, thus restoring Th17/Treg balance, improving airway inflammation and reducing asthma symptoms.

## Background

Asthma is a common, chronic inflammatory disease of the airways characterized by variable and recurring symptoms, reversible airflow obstruction, bronchospasm and hyper-responsiveness (AHR) [1]. With the development of biopsies and bronchoalveolar lavage fluid (BALF) collection technology in the late 1980s, it is now recognized that the common reason for airway obstruction in asthma is chronic airway inflammation [2]. Common symptoms include repeated episodes of wheezing, coughing, shortness of breath, tightness of the chest and symptoms previously described by the National Institutes of Health. Many patients with asthma experience exacerbations at night and/or early in the morning. The most prevalent form of inflammation is allergic pulmonary inflammation, which is initiated by exposure to inhaled allergens and resultant allergen-specific immune responses. As many individuals are exposed to the same inhaled allergens, but not all suffer from asthma, it is essential to determine the pathogenetic pathways of this chronic inflammatory disease. These pathways have been seen in the murine model of asthma.

Many studies have demonstrated a crucial role for T-lymphocytes and the cytokines produced by T-cells in the development of allergic asthma [3, 4]. With the development of the airway inflammation hypothesis, the disturbed balance of the T-helper (Th1/Th2) ratio has become a paradigm in asthma pathogenesis [5]. In addition, the discovery of the mutual inhibitory effects of Th1 and Th2 cells in mice [6] prompted the postulation that an imbalance between these two branches of the immune response would underlie Th1-mediated autoimmune diseases as well as Th2-mediated allergic diseases, including asthma [7]. It has been reported that in conjunction with increasing numbers of Th2 cells, there is an increase in the amount of interleukin 4 (IL-4) secreted, a cytokine that plays a major role in airway allergic inflammation [8]. It is well known that Th1/Th2 lymphocytes play an important role in the initiation, progression and persistence of allergic diseases including asthma. Little is known of the immunoregulatory mechanisms that determine the susceptibility to, the severity of, and the persistence of asthma. Due to its failure to adequately explain many (pre)clinical observations, many researchers consider the imbalance of Th1/Th2 in the mechanism underlying inflammatory and autoimmune disease to be an antiquated paradigm [9].

Currently, T-regulatory (Treg) cells have been recognized as crucial immunoregulatory cells, capable of suppressing Th1- and Th2-mediated adaptive immune responses in a cell contact-dependent fashion [10]. Theoretically, Treg cells may interfere with the development of allergic diseases at different stages, such as allergic sensitization, progression to allergic inflammation, airway remodeling and AHR as well as persistence of disease manifestations. Treg cells expressing CD25 on CD4 + cells, which were discovered in 1995 by Japanese scholars, can negatively regulate activation and proliferation of CD4+ or CD8+ T cells. Treg cells also inhibit the proliferation of naive T cells and memory T cells [11]. Recent studies have also found coexistence of Th1/Th2 and Th17/Treg imbalances in patients with allergic asthma [12]. Th17 cells are a specific T functional cell group, that play key roles in mediating autoimmunity, inflammation and mucosal host defense against pathogens [13]. Th17 cells are largely defined by their eponymous cytokines, IL-17A and IL-17 F, that are pro-inflammatory by virtue of their direct and indirect effects on neutrophil recruitment [14]. Whilst Th17 cells alone do not cause eosinophilic airway inflammation, the IL-23-Th17 cell axis enhances Th2 cell mediated eosinophilic airway inflammation [15]. A novel member of the IL-12 cytokine family, IL-23 has been identified as playing an important pathogenic role in chronic inflammation. It has been reported that IL-23 has effects on eosinophilic recruitment and is crucial for the maintenance of Th17 cells [16].

Recent studies have found that Th17 immunity contributes to the systemic immune responses in allergic asthmatic patients. Individually, neither IL-17 nor ongoing Th2 responses were sufficient to confer AHR. However, they act synergistically to promote neutrophil recruitment, eosinophil recruitment and AHR [17]. The Forkhead box P3 (Foxp3) transcription factor is the key driver of Treg cells differentiation and immunosuppressive function. Foxp3+ Treg cells have essential roles in the maintenance of immune homeostasis and in regulation of effector T cell responses [18]. Foxp3+ Treg cells co-express retinoic acid receptor-related orphan receptor (ROR) γt (in mice) and secrete high levels of IL-17 *ex vivo* [16, 19, 20]. In the absence of a second signal from pro-inflammatory cytokines, Foxp3 can inhibit RORγt function and drive Treg differentiation [21]. When the cell receives a signal from a proinflammation cytokine, such as IL-6, Foxp3 function is inhibited and the Th17 differentiation pathway is induced [21, 22].

A traditional Chinese medicine (TCM) recipe, Pingchuan Formula (PCF), was first proposed by Professor Yu Jianer in 1999. This formula consists of several herbs, including ephedra, almond, perillage semen, raphani semen,, earthworm, peach kernel, scutellaria baicalensis and liquorice. It may regulate the movement of Qi in body, eliminate sputum and promote blood circulation to relieve the symptoms of asthma [23]. Previous studies have indicated that PCF can improve chronic airway inflammation and inhibit airway remodeling bycorrecting the inbalance of Th1/Th2 [24]. However, it has remains unknown how PCF treatment regulates Treg/Th17 cells and their associated cytokines. The current study was performed to investigate the effect of PCF on the balance of Treg/Th17 in an ovalbumin (OVA)-induced asthma mice model with dexamethasone (DEX) as a positive control. Airway inflammation and expression of related transcriptional factors Foxp3 and RORγt in lung tissue were detected in asthmatic mice treated with PCF or DEX over different time periods.

## Methods

### Animals

A total of 80 male BALB/c mice, 5 weeks old, 16-19 g in weight, were purchased from Shanghai Super-B&K Laboratory Animal Corp. Ltd. (grade SPF, production license number: SCXK2008-0016). The animals were housed in a temperature-controlled room at 21–23 °C and maintained on a 12 h light: 12 h dark cycle. All experimental procedures were approved by the Animal Care and Use Sub-committee of the Shanghai Traditional Chinese Medicine Hospital. The mice were housed in macrolon cages in a laminar flow cabinet. All mice were provided with food and water *ad libitum*.

### Mice grouping

Eighty mice were randomly divided into 4 groups: control (CON) group (n = 20), model (MDL) group (asthma induced, no treatment, n = 20), DEX group (asthma induced, DEX treatment for 7 or 28 d, n = 20) and PCF group (asthma induced, PCF treatment for 7 or 28 d, n = 20).

### Model preparation

The mouse asthma model was established as previously described [25] with some modifications. Just before use, a total of 10 g OVA Shanghai Chun Hui Chemical Co., batch number: 20120418) and 1 g aluminum hydroxide were dissolved in 100 ml distilled water to form 10 % sensitizing liquid. Except for the CON group, animals were treated with two intraperitoneal (i.p) injections of 50 ml/kg OVA, spaced 2 weeks apart. Twenty four hours after the last sensitization, the sensitized groups were challenged with 5 % OVA nebulization, 40 min each time, once a day for a total of 1 week. Mice in the CON group were i.p injected and inhaled an equal amount of distilled water.

### Administration of drugs by gavage

The PCF (provided by Shanghai Hospital of TCM) was prepared to 5.33 g/ml concentration as previously described [24]. This was by adding 13 times the volume of water, boiling it and then simmering for 30 min. The liquid was than filtered out and 11 times the volume of water was added for a second round of boiling as before. The two filtrates obtained from boiling were mixed and concentrated, an appropriate amount of 95 % ethanol (calculated according to the formula V * 60/ (95 -60) was added, and refrigerated for 24 h. Then the filtrate was concentrated into liquid extracts. The composition of PCF is as follows: Honey Licorice (9 g); Scutellaria Baicalensis Georgi (9 g); Honey Perilla Seed (9 g); Semen Raphani (9 g); Bitter Apricot Seed (9 g); Peeled Peach Kernel (9 g); Honey Ephedra (6 g); Earthworm (9 g). The estimated chemical formula is therefore based upon the following information from According to the Pharmacopoeia of People’s Republic of China: (2010 edition, published by China Pharmaceutical Science and Technology Press). Honey Herba Ephedraev (after desiccation), contains no less than 0.80 % of ephedrine hydrochloride and pseudoephedrine hydrochloride in weight. Scutellaria baicalensis (after desiccation), contains no less than 9 % baicalin. Perilla Seed (after desiccation) contains not less than 0.25 % of rosmarinic acid, which is the main component. Semen Raphani, contains no less than 0.40 % of sinapine (calculated by sinapine thiocyanate instead), which is the main component. The main component of earthworm is protein, and lysine, leucine, valine were used as reference substances in content determination. The main component of Bitter Apricot Seed is laetrile (no less than 2.4 %). The main component of Peeled Peach Kernel is laetrile (no less than 2 %). Honey Licorice contains liquiritin (no less than 0.50 %) and glycyrrhizic acid (no less than 1 %).

DEX (Shanghai Xinyi Co., Ltd., batch number; 110302) was prepared to a concentration of 0.075 mg/ml. Mice in the CON and MDL groups were provided with distilled water whilst mice in the DEX and PCF groups were fed 20 ml/kg DEX or PCF, respectively. Mice were observed and weighed daily to ensure the correct dose of drug was administered. The weight was recorded periodically. All groups were divided into two and sacrificed at either 7 or 28 d of treatment, respectively and specimens of bronchoalveolar lavage fluid (BALF), blood and lung tissue collected.

### Assessment of clinical symptoms of asthma

The clinical signs of asthma in the mice were investigated at day 7 after OVA challenge. These symptoms included panic and easy irritation, cold aversion and tendency to clump together, abdominal muscle spasm, ruffled hair, oral and nasal cyanosis, and shortness of breath. Each symptom present in a mouse was scored 1 or 0 if not present. A total score of 6 meant that all symptoms were present.

### Differential cell counts in bronchoalveolar lavage fluid (BALF)

Mice were killed, the chest opened with 22 g catheter intubation, the right main bronchus ligated, and the mice douched 3 times with 0.3 ml cold phosphate buffered saline (PBS) [French PBS buffer solution (pH7.2 ~ 7.4)]. The liquid was then siphoned and immediately placed on ice. The BALF was filtered through sterile gauze to remove mucous strands, centrifuged (German Eppendorf Centrifuge Model: 5804R) at 200 g for 5 min at 4 °C and assayed for differential cell counts including eosinophils (EOS) and neutrophils (NE) using an enzyme-linked immunosorbent assay (ELISA) kit as per the manufacturer’s instructions (mouse Eotaxin ELISA Kit and GCP-2 ELISA Kit, RD, US).

### Immunological assays

The concentrations of IL-6, IL-17, IL-23 and TGF-β in BALF were measured using sandwich ELISA kits (ELISA Kit R&D Systems, Minneapolis, MN, USA) according to the manufacturer’s instructions.

### Hematoxylin and Eosin (H&E) staining of lung tissue

The upper lobe of the right lung was removed and placed into a cryovial. 10 % Formalin was added to the tube to fix the lung and it was then prepared for biopsy sectioning. Paraffin-embedded lung sections (Microtome Leica RM2235, 5 μm thick) were stained with hematoxylin and eosin(H&E) and lung structure evaluated by an experienced pathologist using microscopic observation (Olympus BX51, Tokyo, Japan).

### Immunohistochemistry (IHC) assay of lung tissue

Immunohistochemical (IHC) staining of lung samples was performed by a pathologist as previously described [24]. Briefly, paraffin-embedded lung sections (5 μm thick) were cut and placed onto silanized glass slides and air dried before being fixed in cold acetone for 10 min. Sections were then rehydrated in PBS for 10 min and blocked in PBS containing 10 % mouse serum for 30 min in a humidified chamber Friocell Germany MMM incubator (37 °C). Both Foxp3 and RORγt were probed with FITC-conjugated rat anti-mouse monoclonal antibodies (anti-Foxp3: 47 kDa, Epitomics, US; anti-RORγt: 52 kDa, Millipore, US) and visualized using confocal microscopy (Olympus Fluoview 500, Tokyo, Japan) with an excitation wavelength of 488 nm and an emission range of510-550 nm. The FITC-conjugated nonspecific rat IgG isotype control was used as a negative control.

### Immunoblot assay

Lung fragments (0.05 g) were homogenized in ice-cold RIPA lysis buffer (Beyotime, China) and protein concentrations determined using the BCA protein Assay Kit (Beyotime, China) with the absorbance read on a Bio-Rad 680 microplate reader (Bio-Rad, USA). Proteins were separated using the 10 % SDS-PAGE method (Mini-protean 3 Dodeca cell, Bio-Rad, Hercules, CA). The protein was then transferred onto a polyvinylidenedifluoride membrane (Bio-Rad, Hercules, CA). A pre-stained marker was utilized to determine the separation of proteins. The membrane was probed with anti-Foxp3 or anti-RORγt monoclonal antibodies, followed by alkaline phosphatase-conjugated secondary antibodies (BD, USA). Protein was visualized using the brightening agent, ECLTM western blotting detection (GE healthcare, No. RPN2106), and protein band intensity was quantified using the Gel-Pro imaging software (Media Cybernetics, Silver Spring, MD).

### Statistics

Statistical analysis was performed with SPSS 18.0 software (SPSS, Chicago, IL). If the data followed a normal distribution and homogeneity of variance, a one way analysis of variance (ANOVA) and least significant difference (LSD) multiple comparison were used. 2 × 2 factorial design analysis was also employed. A value of P < 0.05 was considered significant.

## Results

### Comparison of survival situation among groups

There was no significant difference in the weight of the animals before being challenged. Following OVA excitation for 7 or 28 d of treatment, the bodyweights of the DEX group were significantly reduced compared with the CON group (P <0.05) (Table 1), while there was no significant difference between PCF and CON groups (P > 0.05).

**Table 1:** Bodyweight of mice in the four groups before challenge and after 7 d of OVA excitation and 28 d of treatment. ($\overline{x}\kern0.5em \pm \kern0.5em s$, g)

Group	Before challenge	Treated for 7 d	Treated for 28 d
	n = 20	n = 10	n = 10
CON	17.10 ± 1.37	22.90 ± 1.10	24.10 ± 1.10
MDL	17.80 ± 0.79	20.90 ± 2.77^a^	23.40 ± 1.58
DEX	17.20 ± 1.23	18.80 ± 0.92^ab^	18.40 ± 1.27^ab^
PCF	17.10 ± 1.20	21.10 ± 1.20^c^	23.30 ± 1.25^c^

^a^compared with CON group, P < 0.05; ^b^ compared with MDL group, P < 0.05; ^c^compared with DEX group, P < 0.05. *OVA* ovalbumin, *CON* control group, *MDL* asthma model group without treatment, *DEX* dexamethasone treated group and *PCF* Pingchuan Formula treated group

The mice in the MDL group showed different degrees of anxiety, muscle twitching, urinary and fecal incontinence, hair fluffing, activity decrease, appetite reduction, oronasal cyanosis and shortness of breath. DEX and PCF groups were roughly similar. Quantification of the symptoms showed that the CON group had a mean score of 0.40 ± 0.50, all of the experimental groups showed significantly higher values (P < 0.05); the MDL group with 5.15 ± 0.59, DEX with 4.40 ± 0.82, and PCF with 4.05 ± 0.76. The values for the DEX and PCF groups were similar but significantly lower than the MDL group (P < 0.05).

### Comparison of pathological changes among groups after 7 d of OVA excitation and 7 or 28 d of treatment

**Fig. 1:**
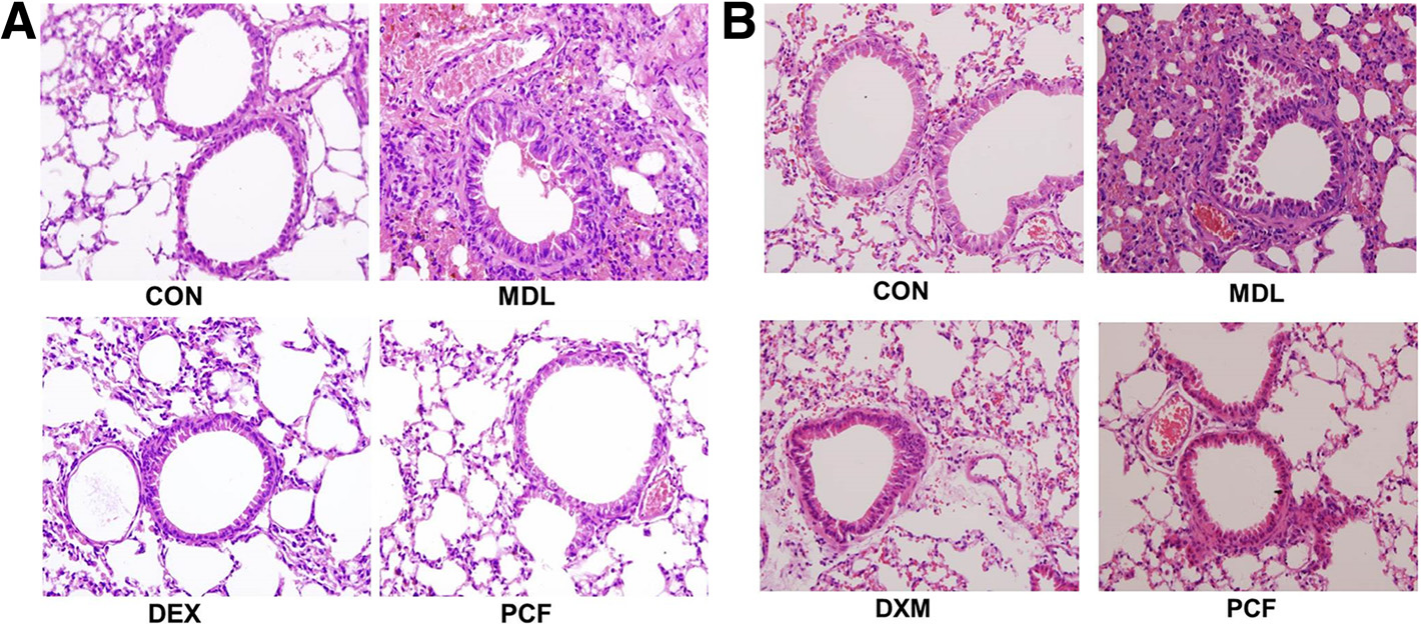
Pathological changes detected using H&E staining in lung tissue after 7 d of OVA excitation and 7 or 28 d of treatment. **a**, H&E stained lung tissues in CON/MDL/DEX/PCF groups after 7 d of excitation with 5 % OVA nebulization (distilled water for CON group) and the daily subsequent treatment by gavage with distilled water for CON and MDL group, 20 ml/Kg of DEX (0.075 mg/ml) for DEX group and 20 ml/Kg of PCF (5.33 g/ml) for PCF group, respectively (n = 10 for each group); **b**, H&E stained lung tissues in CON/MDL/DEX/PCF groups after 7 d of excitation with 5 % OVA nebulization (distilled water for mice CON group) and 28 d treatment by gavage the same as in panel A (n = 10 for each group)

Mice in the CON group had smooth, intact bronchial structure and bronchial epithelial cells were arranged in neat rows. The thickness of the bronchus was normal. There was no inflammatory infiltration around the bronchus. The lumen and peritubular areas had no obvious abnormalities. The MDL group showed bronchial deformation, narrow in diameter, wall structure damage and a large number of inflammatory exudate in the lumen. Bronchial epithelial cells were disordered; goblet cells were hyperplastic and thickened; the bronchus cavity had a chrysanthemum-shaped change. There was infiltration of inflammatory cells around the bronchus, such as eosinophils / neutrophils. There were a large number of inflammatory cell infiltration and accumulation in the perivascular area. The overall condition in the DEX and PCF groups improved slightly compared with the MDL group. The bronchial structure in both the DEX and PCF groups was well-preserved, and cells were arranged in neat rows and the diameter was normal. There was a small amount of inflammatory exudate in the trachea and perivascular area (Fig. 1).

### The difference of differential cell counting in BALF

**Fig. 2:**
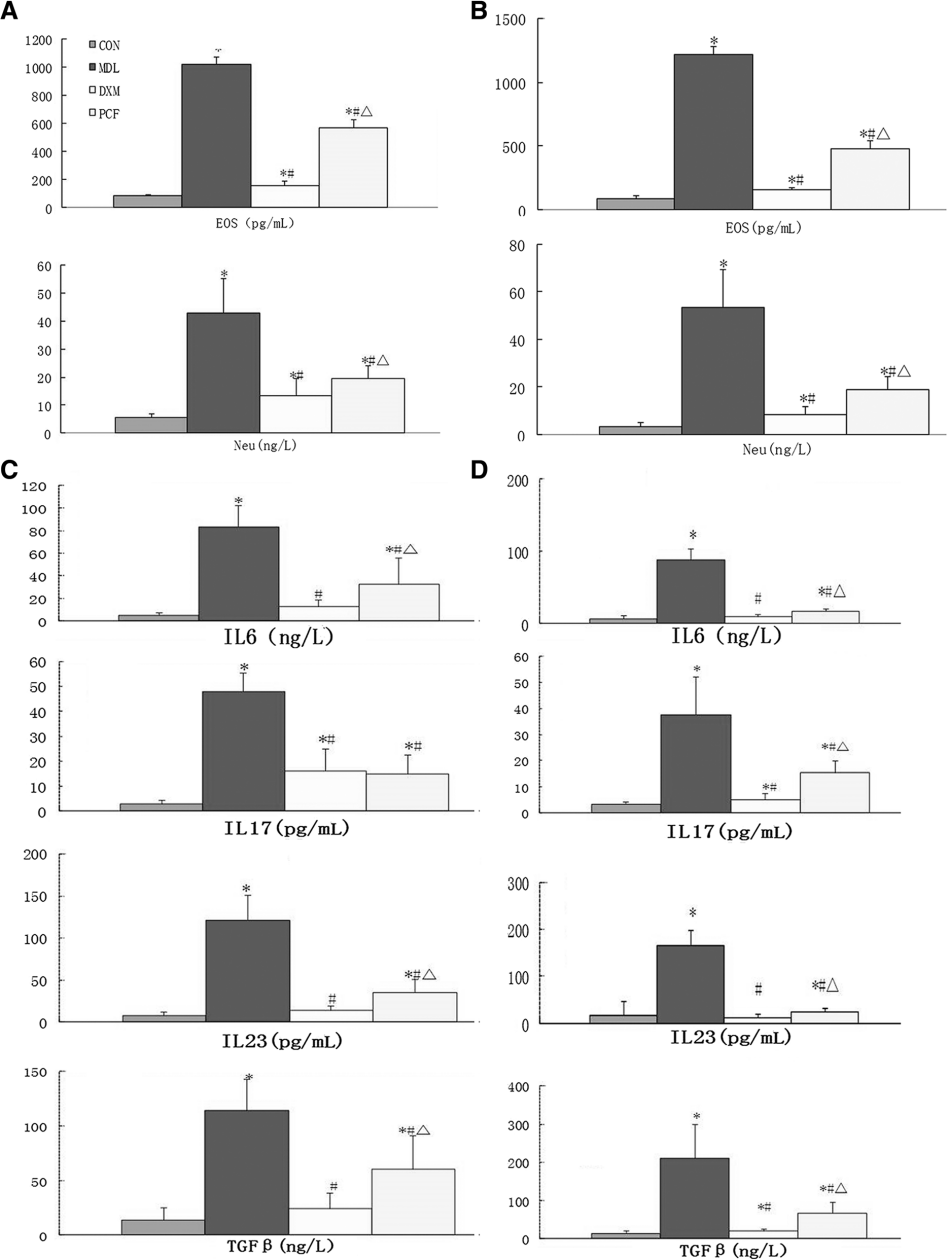
EOS and NE cell levels and Treg/Th17 cytokines levels in BALF after 7 d of OVA excitation and 7 or 28 d of treatment. **a**, EOS and NE cell levels detected using ELISA in BALF of CON/MDL/DEX/PCF groups after 7 d of excitation and treatment (n = 6 for each group); **b**, EOS and NE cell levels detected using ELISA in BALF of CON/MDL/DEX/PCF groups after 7 d of excitation and 28 d of treatment (n = 6 for each group). **c**, IL-6, IL-17, IL-23 and TGF-β levels detected using ELISA in BALF of CON/MDL/DEX/PCF groups after 7 d of excitation and treatment (n = 10 for each group); **d**, IL-6, IL-17, IL-23 and TGF-β levels detected using ELISA in BALF of CON/MDL/DEX/PCF groups after 7 d of excitation and 28 d treatment (n = 10 for each group). *compared with CON group,P < 0.05; # compared with MDL group,P < 0.05; Δ compared with DEX group,P < 0.05

EOS were the major type of cells in BALF in the acute and sub-acute stages (day 7 and day 28). EOS and NE cells were detected using ELISA in BALF of mice (n = 6 for each group) which was administrated 7 or 28 d of PCF/DEX. The data showed that sensitized mice in the MDL group had extremely high levels of EOS and NE cells. We also found that PCF groups had decreased EOS and NE cells in BALF (Fig. 2).

### Comparison of Treg / Th17 cytokines IL-6, IL-17, IL-23 and TGF-β among groups

**Fig. 3:**
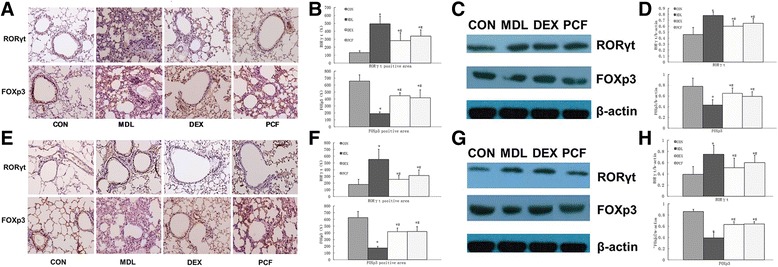
Expression of asthmatic airway RORγt and Foxp3 protein after 7 d of OVA excitation and 7 or 28 d of treatment. **a**, Immunohistochemistry assay of RORγt and Foxp3 in lung tissues of CON/MDL/DEX/PCF groups after 7 d of excitation and treatment (n = 10 for each group); **b**, quantitative analysis of panel (**a**); **c**, immunoblot assay of RORγt and Foxp3 in lung tissues of CON/MDL/DEX/PCF groups after 7 d of excitation and treatment (n = 10 for each group); **d**, quantitative analysis of panel (**c**); **e**-**h**, data of 28 d of treatments parallel to panel A-D (n = 10 for each group). *compared with CON group,P < 0.05; # compared with MDL group,P < 0.05; Δ compared with DEX group,P < 0.05

Groups were compared using analysis of variance (ANOVA) and LSD (Fig. 3). After 7 d of OVA excitation and 7/28 d of treatment, the MDL group had higher levels of all cytokines compared with the PCF and DEX groups (P <0.05), whilst the levels of all cytokines in the PCF and DEX groups were not significantly different.

### Expression of asthmatic airway RORγt and Foxp3 protein

Following 7 d of OVA excitation and 7 or 28 d of treatment, RORγt expression was higher in the MDL group compared with the CON group, however Foxp3 was significantly lower in the MDL group compared with the CON group (P < 0.05, Fig. 3). Both the PCF and DEX groups had significantly lower RORγt levels but significantly higher Foxp3 levels compared with the MDL group (P < 0.05). There was no significant difference in RORγt and Foxp3 levels between the PCF and DEX groups.

### Factorial analysis of treatment and time

The DEX and MDL groups were compared using a 2 × 2 factorial design (untreated/treated, treatment for 7/28 days) in IL-6, IL-17, IL-23, TGF-β levels and RORγt, Foxp3 expression. The results are presented in Table 2 and show that improvements of IL-23 and TGF-β levels by DEX treatment were associated with time. The difference of IL-23/TGF-β concentrations between DEX (28d -7d) and MDL (28d-7d) is presented in Table 3.

**Table 2:** Factorial analysis [2 × 2 factorial design (untreated/treated, treatment for 7/28 d)] of IL-6, IL-17, IL-23, TGF-β levels and RORγt, Foxp3 expression between the MDL and DEX groups

Variation	Indicator	*SS*	*df*	*MS*	*F*	*P*
Group (difference between MDL and DEX group)	IL-6	56480.11	1	56480.11	346.92	<0.001
	IL-17	10396.43	1	10396.43	118.14	<0.001
	IL-23	168584.56	1	168584.56	329.16	<0.001
	TGF-β	197694.57	1	197694.57	88.65	<0.001
	RORγt	0.45	1	0.45	27.11	<0.001
	Foxp3	0.52	1	0.52	66.39	<0.001
Time (difference between 7 d and 28 d treatment)	IL-6	0.11	1	0.11	0.00	0.979
	IL-17	1169.06	1	1169.06	13.29	0.001
	IL-23	4214.55	1	4214.55	8.23	0.007
	TGF-β	21824.31	1	21824.31	9.79	0.003
	RORγt	0.04	1	0.04	2.26	0.141
	Foxp3	0.01	1	0.01	1.36	0.251
Group *Time	IL-6	220.59	1	220.59	1.36	0.252
	IL-17	0.20	1	0.20	0.00	0.962
	IL-23	4784.55	1	4784.55	9.34	0.004
	TGF-β	25241.88	1	25241.88	11.32	0.002
	RORγt	0.01	1	0.01	0.49	0.490
	Foxp3	0.00	1	0.00	0.04	0.841
SE	IL-6	5860.97	36	162.81	--	--
	IL-17	3167.92	36	88.00	--	--
	IL-23	18438.26	36	512.17	--	--
	TGF-β	80284.78	36	2230.13	--	--
	RORγt	0.59	36	0.02	--	--
	Foxp3	0.28	36	0.01	--	--

A P value <0.05 was considered statistically significant

*MDL* asthma model group without treatment, *DEX* dexamethasone treated group, *IL-6* interleukin-6, *IL-17* interleukin-17, *IL-23* interleukin-23, *TGF-β* transforming growth factor-beta, *RORγt* RAR-related orphan receptor gamma, *Foxp3* forkhead box P3**

*Group *Time means that both factor "Group" and "Time" are considered simultaneously

**Table 3:** Difference of IL-23 and TGF-βlevels in BALF between DEX and MDL groups after DEX or distilled water treatment for 7 or 28 d

	IL-23				TGF-β			
Group	Time		Mean	28d-7d	Time		Mean	28d-7d
	7d	28d			7d	28d		
MDL	121.70	164.11	142.91	42.41	114.31	211.26	162.79	96.95
DEX	13.74	12.39	13.07	-1.35	23.94	20.42	22.18	-3.52
Mean	67.72	88.25	--	20.53	69.13	115.84	--	46.72
DEX - MDL	-107.96	-151.72	-129.84	--	-90.37	-190.84	-140.61	--

*MDL* asthma model group without treatment, *DEX* dexamethasone treated group, *BALF* bronchoalveolar lavage fluid, *IL-23* interleukin-23, *TGF-β* transforming growth factor-beta

The PCF and MDL groups were also compared using a 2× 2 factorial design (Table 4). The results indicated that improvements of IL-23 and TGF-β by PCF treatment were associated with time of treatment. The difference of IL-23/TGF-β concentrations between PCF (28d -7d) treatment and MDL (28d-7d) treatment is presented in Table 5. The data (Table 3 and Table 5) shows that PCF treatment and increase in the length of treatment significantly decreased IL-23 compared with DEX treatment.

**Table 4:** Factorial analysis [2 × 2 factorial design (untreated/treated, treatment for 7/28 d)] of IL-6, IL-17, IL-23, TGF-β levels and RORγt, Foxp3 expression between the MDL and PCF groups

Variation	Indicator	*SS*	*df*	*MS*	*F*	*P*
Group (difference between MDL and PCF group)	IL-6	38352.38	1	38352.38	131.94	<0.001
	IL-17	7712.34	1	7712.34	89.54	<0.001
	IL-23	128013.36	1	128013.36	227.20	<0.001
	TGF-β	99794.91	1	99794.91	38.07	<0.001
	RORγt	0.21	1	0.21	16.11	<0.001
	Foxp3	0.42	1	0.42	57.65	<0.001
Time (difference between 7 d and 28 d treatment)	IL-6	377.41	1	377.41	1.30	0.262
	IL-17	261.65	1	261.65	3.04	0.090
	IL-23	2497.38	1	2497.38	4.43	0.042
	TGF-β	26216.14	1	26216.14	10.00	0.003
	RORγt	0.02	1	0.02	1.42	0.241
	Foxp3	0.00	1	0.00	0.08	0.786
Group *Time	IL-6	1198.32	1	1198.32	4.12	0.050
	IL-17	308.50	1	308.50	3.58	0.066
	IL-23	7075.54	1	7075.54	12.56	0.001
	TGF-β	20936.21	1	20936.21	7.99	0.008
	RORγt	0.00	1	0.00	0.08	0.783
	Foxp3	0.02	1	0.02	2.85	0.100
SE	IL-6	10464.63	36	290.68	--	--
	IL-17	3100.86	36	86.16	--	--
	IL-23	20284.27	36	563.45	--	--
	TGF-β	94369.00	36	2621.36	--	--
	RORγt	0.46	36	0.01	--	--
	Foxp3	0.26	36	0.01	--	--

A P value <0.05 was considered statistically significant

*MDL* asthma model group without treatment, *PCF* Pingchuan Formula treated group, *IL-6* interleukin-6, *IL-17* interleukin-17, *IL-23* interleukin-23, *TGF-β* transforming growth factor-beta, *RORγt* RAR-related orphan receptor gamma, *Foxp3* forkhead box P3

*Group *Time means that both factor "Group" and "Time" are considered simultaneously

**Table 5:** Difference of IL-23 and TGF-βlevels in BALF between PCF and MDL groups after PCF or distilled water treatment for 7 or 28 d

	IL-23				TGF-β			
Group	Time		Mean	28d-7d	Time		Mean	28 d-7 d
	7 d	28 d			7 d	28 d		
MDL	121.70	164.11	142.91	42.41	114.31	211.26	162.79	96.95
PCF	35.16	24.36	29.76	-10.80	60.16	65.61	62.89	5.45
Mean	78.43	94.24	--	15.81	87.24	138.44	--	51.20
PCF - MDL	-86.54	-139.75	-113.15	--	-54.15	-145.65	-99.90	--

*MDL* asthma model group without treatment, *PCF* Pingchuan Formula treated group, *BALF* bronchoalveolar lavage fluid, *IL-23* interleukin-23, *TGF-β* transforming growth factor-beta

## Discussion

Whilst glucocorticoid treatment is the primary treatment choice for asthma patients, traditional Chinese medicine (TCM), used in Asia for centuries, is beginning to play a role in Western health care as a complementary and alternative medicine, especially for mild-to-moderate asthma. There is increasing scientific evidence supporting the use of TCM for asthma treatment. Chinese herbal medicines are one of the major components in TCM practice and are prescribed in hospitals in China as either monotherapy or complementary therapy to conventional Western therapy. In the present study, PCF successfully decreased the levels of cytokines and restored Treg/Th17 balance in the asthma mouse model, thus improving airway inflammation and reducing asthma symptoms. These findings are consistent with previous reports investigating the efficacy of herbal interventions for asthma, whereby TCM herbal significantly increased forced expired volume in 1 s (FEV1) levels (P < 0.05) in asthma patients compared with controls [26]. Various different TCM herbal remedies have also been shown to improve inflammation indices and cytokines [27–29]. Several clinical studies have revealed that TCM herbal remedies are of value for the treatment of asthma and are of particular benefit in that TCM herbal remedies rarely cause adverse reactions and side effects. In accordance with previous studies investigating other TCM herbal remedies for asthma, namely antiasthma herbal medicine intervention (ASTHMI) [30], PCF treatment in the current study induced weight gain.

It can be difficult to unravel the active ingredients of some TCM herbal remedies that are responsible for the therapeutic effects because of the complex ingredients and the intended interaction of those ingredients. A study on another TCM herbal remedy for asthma suggested that Cynodon dactylon (Linn.) contained scopoletin, a coumarin from plant roots, as an active ingredient from densitometry analyses [27]. The long list of potential active ingredients in PCF include, ephedrine hydrochloride, pseudoephedrine hydrochloride, baicalin, rosmarinic acid, sinapine, laetrile, liquiritin and glycyrrhizic acid. Of these the most obviously related to asthma treatment is ephedrine hydrochloride, which is an asthma medication, classed as a bronchodilator, which was commonly used up to the 1950s [31]. This active ingredient might partly explain the reduction of asthma symptoms in the treated mice in this study, but the interaction of the other active ingredients might have to be unraveled to explain the decreased the levels of cytokines and restored Treg/Th17 balance. These results support other studies that have investigated the role of PCF and inflammation where mice treated with PCF were found to have a corrected imbalance of Th1/Th2 [24], decreased IL-5 and an adjusted balance of oxidants/antioxidants [32], and children with asthma have been shown to have reduced IL-4 and IL-13 and increased interferon (INF)-γ [33].

Normal bronchial mucous glands and goblet cells secrete only a small amount of mucus to keep the airway moist. When the airway has been inflamed, including mucosal edema, mucus secretion, increased permeability of the capillary wall, serous will be exuded. At this time, sputum containing red blood cells, white blood cells, macrophages, and other fibrins will be exuded. Mucus, inhalation of dust and some tissue damage will be discharged with a cough [24]. From the Western perspective, sputum is a pathological product of airway inflammation. In TCM however, phlegm formed due to a disorder in body fluid metabolism, is closely related with the functioning of the lung. The purpose of compatibility of PCF is to recover the pulmonary function and regulate the pulmonary qi. Thereby, it can relieve asthma and reduce phlegm.

This study revealed that in asthma model, pro-inflammatory cytokines IL-6, IL-17, IL-23 and TGF-β were increased. The expression of transcriptional factor RORγt was increased exponentially but Foxp3 was significantly reduced. These data indicate an increase in Th17 cells and a decrease in Treg cells, suggesting an imbalance in Treg/Th17, consistent with previous studies [34].

In the present study, compared with the CON group, the MDL group lost bodyweight and exhibited symptoms after 7 d of excitation. Lung biopsy revealed the airway structure was damaged, including bronchial deformation, diameter narrowing. And there was a lot of intraluminal inflammatory infiltration, perivascular inflammatory cell infiltration and accumulation. Inflammatory cytokines increased. The expression of RORγt and Foxp3 was disturbed with an increase in RORγt and a decrease in Foxp3. These differences were statistically significant, indicating that MDL was a successful model group. All these indexes in the PCF group were improved, and were statistically significant. The results indicate that PCF has an effect on the asthma model group. It can reduce IL-6, IL-17, IL-23, TGF-β and RORγt expression, and elevate Foxp3 expression. Compared with the DEX group, PCF increased bodyweight and was not inferior with the DEX group in terms of the regulation of IL-6, IL-17, TGF-β, RORγt and Foxp3.

The PCF and DEX groups were compared with the MDL group in 2 × 2 factorial analysis. The difference between the PCF group and the DEX group in the expression of IL-6, IL-17, RORγt and Foxp3 was simply due to processing factors. The PCF group and the DEX group both had cumulative effects on IL-23 and TGF-β. The PCF group had better accretion than DEX on IL-23.

The current study has several limitations, indicating the need for future research in the investigation of TCM herbal remedies for the treatment of asthma. Of importance is the fact that this research utilized an animal model of asthma. Whilst the murine asthma model is often and regularly used in research, relating these findings to clinical settings remains difficult. Further clinical investigations of PCF treatment for asthma are required to verify and support the findings of the current study. This was a relatively short-term study and it would be beneficial to determine the effectiveness of PCF in the long-term treatment of asthma. A long-term study would provide information as to any possible long-term side effects of PCF. In addition, we did not include a control group that had been treated with PCF, it would be interesting in future studies to investigate the action of PCF on the healthy lung.

## Conclusions

This study reveals that PCF can downregulate RORγt and elevate Foxp3 expression, reduce IL-6, IL-17, IL-23, TGF-β in BALF, thus restore the balance of Th17/Treg, improve airway inflammation and reduce asthma symptoms. This TCM, PCF leads to less loss of body weight in mice and more accretion of IL-23.

## Abbreviations

PCF: Pingchuan formula
HE: Hematoxylin and eosin
ELISA: Enzyme-linked immunosorbent assay
AHR: Asthma is a common, chronic inflammatory disease of the airways characterized by variable and recurring symptoms, reversible airflow obstruction, bronchospasm and hyperresponsiveness
CON: Control group
MDL: Model group
DEX: Dexamethasone group
PBS: Phosphate buffered saline
EOS: Cell counts including eosinophils
NE: Neutrophils.
